# Genetic diversity and differentiation of the rhesus macaque (*Macaca mulatta*) population in western Sichuan, China, based on the second exon of the major histocompatibility complex class II DQB (*MhcMamu-DQB1*) alleles

**DOI:** 10.1186/1471-2148-14-130

**Published:** 2014-06-14

**Authors:** Yong-Fang Yao, Qiu-Xia Dai, Jing Li, Qing-Yong Ni, Ming-Wang Zhang, Huai-Liang Xu

**Affiliations:** 1 College of Animal Science and Technology, Sichuan Agricultural University, Ya´an 625014, China; 2 College of Life Sciences, Sichuan University, Chengdu 610064, China; 3 Experimental Animal Engineering Center/National Experimental Macaque Reproduce Laboratory, Sichuan Agricultural Universiy, Ya′an 625014, China

**Keywords:** Genetic diversity, Genetic differentiation, *Macaca mulatta*, Major histocompatibility complex, Trans-species evolution

## Abstract

**Abstracts:**

**Background**

Rhesus macaques living in western Sichuan, China, have been separated into several isolated populations due to habitat fragmentation. Previous studies based on the neutral or nearly neutral markers (mitochondrial DNA or microsatellites) showed high levels of genetic diversity and moderate genetic differentiation in the Sichuan rhesus macaques. Variation at the major histocompatibility complex (MHC) loci is widely accepted as being maintained by balancing selection, even with a low level of neutral variability in some species. However, in small and isolated or bottlenecked populations, balancing selection may be overwhelmed by genetic drift. To estimate microevolutionary forces acting on the isolated rhesus macaque populations, we examined genetic variation at *Mhc-DQB1* loci in 119 wild rhesus macaques from five geographically isolated populations in western Sichuan, China, and compared the levels of MHC variation and differentiation among populations with that previously observed at neutral microsatellite markers.

**Results**

23 *Mamu-DQB1* alleles were identified in 119 rhesus macaques in western Sichuan, China. These macaques exhibited relatively high levels of genetic diversity at *Mamu-DQB1*. The Hanyuan population presented the highest genetic variation, whereas the Heishui population was the lowest. Analysis of molecular variance (AMOVA) and pairwise *F*_ST_ values showed moderate genetic differentiation occurring among the five populations at the *Mhc-DQB1* locus. Non-synonymous substitutions occurred at a higher frequency than synonymous substitutions in the peptide binding region. Levels of MHC variation within rhesus macaque populations are concordant with microsatellite variation. On the phylogenetic tree for the rhesus and crab-eating macaques, extensive allele or allelic lineage sharing is observed betweenthe two species.

**Conclusions**

Phylogenetic analyses confirm the apparent trans-species model of evolution of the *Mhc-DQB1* genes in these macaques. Balancing selection plays an important role in sharing allelic lineages between species, but genetic drift may share balancing selection dominance to maintain MHC diversity. Great divergence at neutral or adaptive markers showed that moderate genetic differentiation had occurred in rhesus macaque populations in western Sichuan, China, due to the habitat fragmentation caused by long-term geographic barriers and human activity. The Heishui population should be paid more attention for its lowest level of genetic diversity and relatively great divergence from others.

## Background

How genetic variation is maintained within populations of endangered species is a central issue in evolutionary and conservation biology [1]. Diversity as a measure of individual variation within a population is widely thought to reflect the number of different genotypes in the population, taking into account their frequencies [2]. Neutral markers are often a measure of genetic diversity, but neutral genetic variations do not always correlate with variations at adaptively important genes [3]. Therefore, the importance of genetic diversity at markers of adaptive significance has been increasingly recognized [4,5]. The multi-gene major histocompatibility complex (MHC) family is found in vertebrates, coding for cell surface glycoproteins and is important in animal conservation due to its role in resisting pathogens [6]. The classical MHC genes of rhesus macaques can be divided into MHC class I and II genes. The MHC class I genes include mainly -A and -B alleles, and MHC class II genes include mainly *-DM*, −*DO*, −*DP*, −*DQ*, and *-DR* alleles. MHC genes are well-known examples of genes of adaptive significance and are particularly relevant to conservation [7]. Consequently, MHC variability is a reflection of the processes that are related to adaptive evolution within and between populations [8], and the generation and maintenance of allelic polymorphisms in the MHC genes is a major topic in evolutionary genetics. The high levels of polymorphisms usually observed at MHC genes are most likely to be maintained by balancing selection, and driven largely by exposure to a diversity of pathogens [9-11], but some studies on MHC variation in natural populations show that balancing selection does not always maintain high levels of MHC variation relative to neutral markers [12,13]. In particular in small populations, the strength of balancing selection on MHC genes may be weak relative to other microevolutionary forces such as genetic drift [6,14]. Genetic drift is the random fluctuation of allele frequencies over time, thus, adaptive alleles may be lost, and deleterious alleles could be fixed in the population. Many natural populations are threatened by intense reduction and fragmentation of habitat, which leads to population isolation, decline in the number and the loss of genetic diversity [15]. The small population size and fixation of deleterious alleles leads to inbreeding depression and reduction of individual fitness, which decreases viability and compromises a population’s evolutionary adaptive potential [16]. The loss of genetic diversity may increase the risk of extinction due to decreased reproductive fitness, decreased adaptive flexibility, and increased disease susceptibility [17]. An important assumption in conservation genetics is that small and isolated populations are more sensitive to genetic drift and inbreeding [18,19]. Clarifying the mechanism that determines genetic variation in small and isolated populations is therefore essential for species conservation [19].

**Figure 1:**
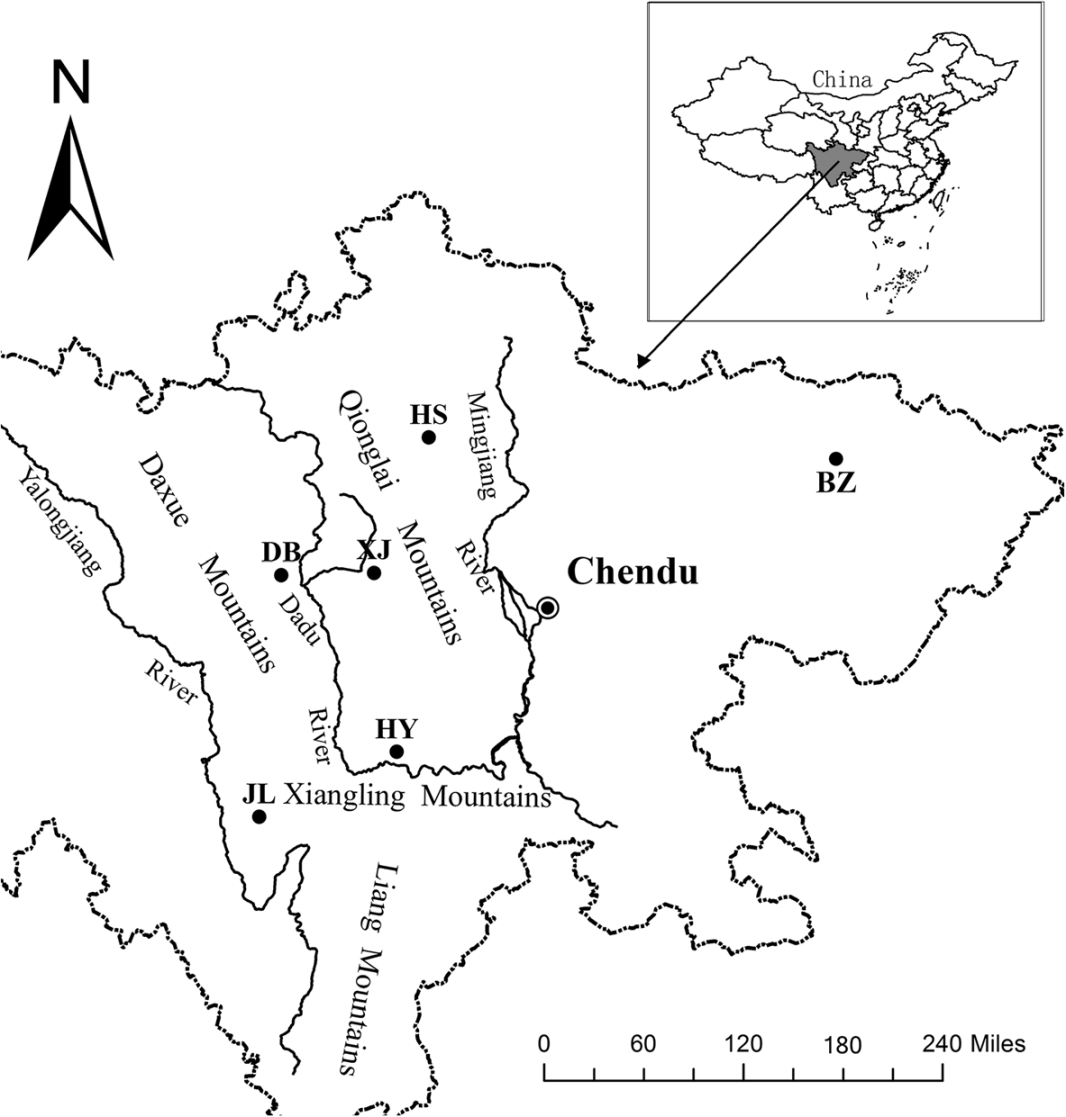
**Distribution of sampling localitys in western Sichuan.** Each filled circle representing a population and filled triangle representing Gongga Mountains (GGM). JL, Jiulong; HY, Hanyuan; DB, Danba; XJ, Xiaojin; HS, Heishui. The map in this figure was produced by author Qiuxia Dai.

Western Sichuan, located along the southeastern edge of the Qinghai-Tibetan Plateau (QTP) and the northern section of Hengduan Mountains, is the global biodiversity hotspots. Affected by the Pliocene uplifting of the QTP, western Sichuan formed a complex and diverse topography with high mountains and deep valleys. The region, from southwest to northeast, has four major mountains, namely, Liangshan, Xiangling, Qionglai, and Mingshan and four large rivers, namely, Jinshajiang, Yalongjiang, Daduhe, Mingjiang and Jialingjiang (Figure 1). Additionally, there are many human settlements and roads in the region. Thus, many wild animals and plants – endangered species in particular – have actually formed fragmented distributions in western Sichuan due to the barrier effect of these natural and man-made impediments. The rhesus macaque (*Macaca mulatta*) is mainly found throughout most of southern Asia crossing eastern Afghanistan, Bangladesh, Bhutan, northern and central India, central and southern China, Lao PDR, Myanmar, Nepal, northern Pakistan, northern Thailand, and Vietnam, and is a endangered species due to the sharp reduction in the number throughout its wider distribution in recent years. This species is listed in CITES Appendix II, Schedule III of the Bangladesh Wildlife (Preservation) Act (1974), Schedule I of Part I of the Indian Wildlife (Protection) Act (amended up to 2002), and Category II of the Chinese Wildlife Protection Act (1989). According to Jiang [20], Chinese rhesus macaques were divided into six subspecies. Rhesus macaques in western Sichuan are classified as a separate subspecies and designated as *M. mulatta lasiotis*, which was isolated from the other five subspecies by human activities and natural barriers (Yangtze River) and has been fragmentized in western Sichuan mainly due to the special topography in this region (Figure 1). Therefore, western Sichuan is a suitable place to explore how microevolutionary forces such as balancing selection and genetic drift maintain the variation pattern of rhesus macaque populations in western China. Understanding evolutionary processes that maintain genetic diversity in natural populations is an essential goal of population genetics [21,22]. Neutral or nearly neutral markers such as mitochondrial or microsatellite DNA are informative for phylogenetic and phylogeographic reconstructions [23]. Genetic diversity analysis using mitochondrial DNA markers showed that significant genetic differentiation had occurred among different isolated populations of Sichuan rhesus macaques [24]. Subsequent microsatellite loci analysis also demonstrated that the genetic differentiation has occurred between a local (Heishui) population and other populations [25]. Another study, using a large dataset of maternally inherited mitochondrial DNA gene sequences and nuclear microsatellite DNA data, revealed two maternal super haplogroups that exist in Chinese rhesus macaques, one in eastern China and another in western China, and further analysis showed that orogenesis likely drove the divergence of western populations in China [26].

The rhesus macaque, cynomolgus macaque, common marmoset and owl monkey are non-human primates that are often used as experimental model animals for biomedical research, such as transplantation studies and vaccine development against measles, Ebola, or other infectious diseases including acquired immune deficiency syndrome (AIDS) [27,28]. For use in immune-related studies, it is necessary that the MHC background of these animals be characterized. Until now, various MHC genes including class I and II have been identified and characterized in different degrees for these non-human primate animals. For example, 14 *Aona-DQB1* alleles were identified that separated the two lineages Aona-DQB1*22 and Aona-DQB1*23 in a group of 19 unrelated owl monkeys (*Aotus nancymaae*) [29]; Otting et al. [30] sequenced exon 2 of the *Mhc-DQB* gene in each of a random panel of 60 non-pedigreed cynomolgous macaques (*Macaca fascicularis*), resulting in the detection of 23 *Mafa-DQB1* alleles that had not previously been published and confirmed the trans-species model of evolution of the *Mhc-DQB* lineages, in which a group of major allele lineages is shared by different species of non-human primates in the phylogeny. Qiu et al. [31] identified 37 *MhcMamu-DQB1* alleles in 105 Chinese rhesus macaques by cloning and sequencing, illustrating a marked allelic polymorphism at *DQB1* in these monkeys. Notably, in recent years more MHC class II *DQB* alleles have been identified and characterized in two closely related monkeys, rhesus and cynomolgous macaques, due to their common use as experimental animal models in biomedical research.

The above work provided us with the inspiration and base to explore the genetic variation patterns of rhesus macaques in western China based on MHC genes with adaptive significance because of their role in pathogen resistance. The aims of this study included the following: 1) to investigate polymorphism levels of MHC class II *DQB1* locus in five isolated populations of rhesus macaques in western Sichuan, China; 2) to estimate the degree of genetic differentiation among or within populations; 3) to determine the relative roles of microevolutionary forces such as balancing selection or genetic drift in populations; and 4) to confirm the trans-species model of evolution of *Mhc-DQB1* lineages between rhesus and cynomolgous macaques.

## Results

### Frequency distribution of *Mamu-DQB1* alleles

A total of 119 rhesus macaque individuals in western Sichuan were typed into 49 genotypes through the polymerase chain reaction (PCR) and single-strand conformation polymorphism (SSCP) analysis based on the polymorphism of the second exon of the *Mamu-DQB1* gene, and then cloning and sequencing of the second exon of the *Mamu-DQB1* gene were conducted for the samples representing the 49 genotypes. Consequently, 23 *Mamu-DQB1* alleles were identified in Sichuan rhesus macaques (Table 1). Each *Mamu-DQB1* sequence obtained was 274 bp (excluding primers) in length and spanned the complete sequence of exon 2. No more than two alleles were detected in each individual, and no stop codon or insertion/deletion was observed in *Mamu-DQB1*. By blast searches in the IPD-MHC Database (http://www.ebi.ac.uk/ipd/mhc/), the 23 allelic sequences were found to be completely identical to some designated alleles, showing that these alleles also existed in other rhesus macaque individuals in previous study.

**Table 1:** **Distribution of allelic frequencies for *Mamu-DQB1 *in five rhesus macaque populations**

**Alleles**	**Total (n = 119)**		**JL (n = 20)**		**HY (n = 28)**		**DB (n = 14)**		**XJ (n = 27)**		**HS (n = 30)**	
	**NA**	**AF**	**NA**	**AF**	**NA**	**AF**	**NA**	**AF**	**NA**	**AF**	**NA**	**AF**
1. Mamu-DQB1*0605	19	7.98	17	42.50	2	3.57	-	-	-	-	-	-
2. Mamu-DQB1*0606	25	10.50	2	5.00	-	-	7	25.00	13	24.07	3	5.00
3. Mamu-DQB1*0607	3	1.26	3	7.50	-	-	-	-	-	-	-	-
4. Mamu-DQB1*0610	3	1.26	1	2.50	2	3.57	-	-	-	-	-	-
5*.* Mamu-DQB1*061101	13	5.46	1	2.50	-	-	5	17.86	1	1.85	6	10.00
6. Mamu-DQB1*061102	2	0.84	-	-	1	1.79	-	-	1	1.85	-	-
7. Mamu-DQB1*061301	1	0.42	-	-	-	-	1	3.57	-	-	-	-
8. Mamu-DQB1*0614	31	13.03	1	2.50	1	1.79	6	21.43	4	7.41	19	31.67
9. Mamu-DQB1*0617	8	3.36	-	-	-	-	-	-	3	5.56	5	8.33
10. Mamu-DQB1*1501	6	2.52	4	10.00	1	1.79	-	-	1	1.85	-	-
11. Mamu-DQB1*1502	4	1.68	-	-	4	7.14	-	-	-	-	-	-
12. Mamu-DQB1*1503	30	12.61	-	-	7	12.50	3	10.71	19	35.19	1	1.67
13. Mamu-DQB1*1601	2	0.84	-	-	2	3.57	-	-	-	-	-	-
14. Mamu-DQB1*1703	29	12.18	-	-	-	-	-	-	4	7.41	25	41.67
15. Mamu-DQB1*170601	3	1.26	-	-	3	5.36	-	-	-	-	-	-
16. Mamu-DQB1*1709	5	2.10	4	10.00	-	-	-	-	-	-	1	1.67
17. Mamu-DQB1*1801	8	3.36	3	7.50	3	5.36	2	7.14	-	-	-	-
18. Mamu-DQB1*1804	5	2.10	-	-	5	8.93	-	-	-	-	-	-
19. Mamu-DQB1*1810	7	2.94	1	2.50	2	3.57	-	-	4	7.41	-	-
20. Mamu-DQB1*1811	23	9.66	2	5.00	15	26.79	2	7.14	4	7.41	-	-
21. Mamu-DQB1*1812	3	1.26	1	2.50	2	3.57	-	-	-	-	-	-
22. Mamu-DQB1*1818	2	0.84	-	-	-	-	2	7.14	-	-	-	-
23. Mamu-DQB1*1819	6	2.52	-	-	6	10.71	-	-	-	-	-	-

Notes: NA, number of alleles; AF, frequency of alleles (Gene frequency shown in the table was the percentage); n, number of individual. Shot line (hyphen) means zero. The bold allelic name showed 100% identity between *Mamu-DQB1* and *Mafa-DQB1nucleotide sequences*. JL, Jiulong; HY, Hanyuan; DB, Danba; XJ, Xiaojin; HS, Heishui. Asterisk* is only a separate symbol between Mamu-DQB1 and allele number in the allele name.

**Figure 2:**
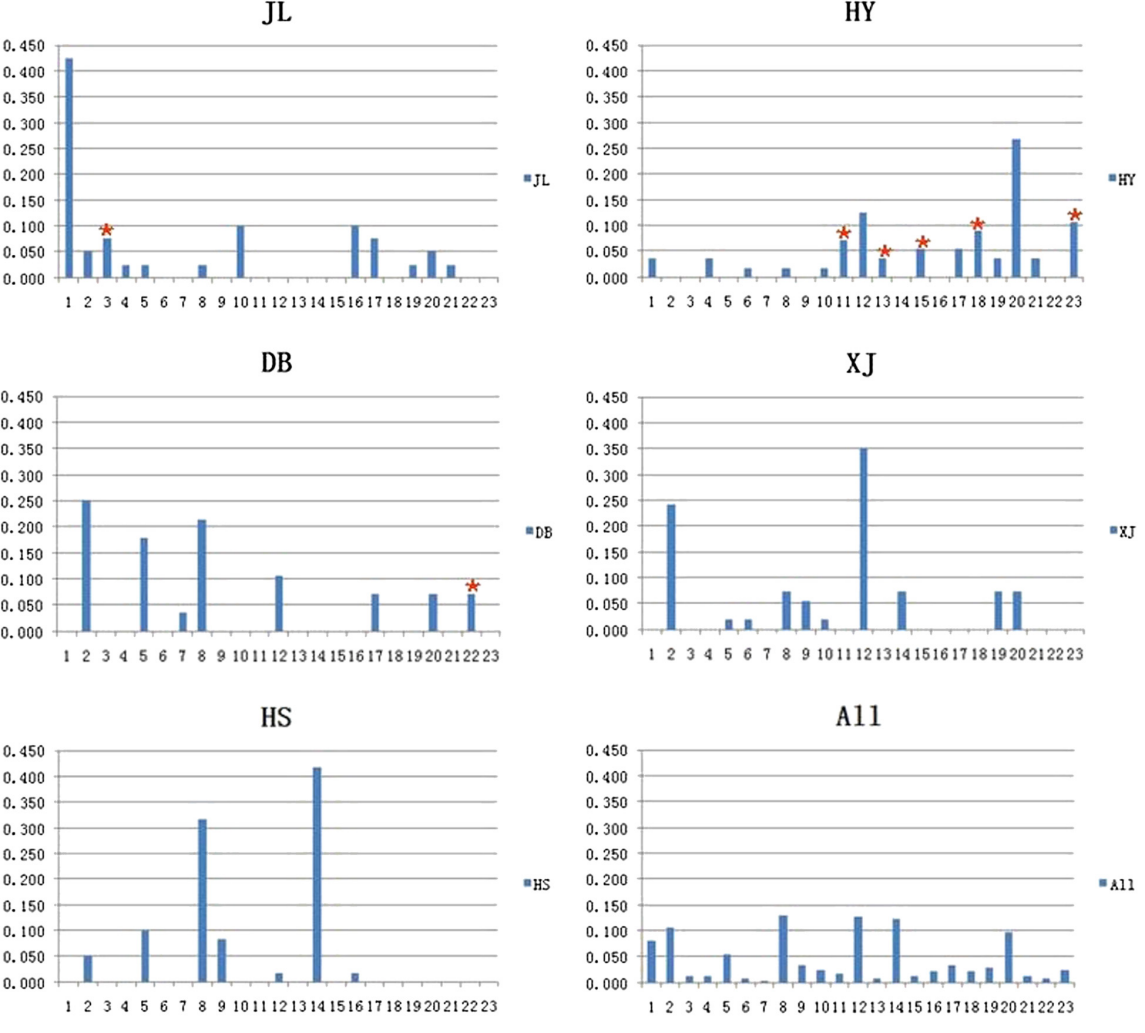
**Histogram of *MhcMamu-DQB1 *frequency distribution in populations.** The y-axis represents the alliic frequency and x-axis represents 23 *MhcMmamu-DQB1* alleles, No. 1–23 presents each allele name in Table 1; JL, Jiulong; HY, Hanyuan; DB, Danba; XJ, Xiaojin; HS, Heishui. Red asterisk (*) represents group-specific alleles in this population.

The number and frequency of the *Mamu-DQB1* alleles varied substantially in five different geographical populations (Table 1 and Figure 2). The highest frequency was Mamu-DQB*0614 among these rhesus macaques, which was found in 31 (13.03%) of the 119 monkeys; the next most common frequency was Mamu-DQB*1503, which was detected in 30 (12.61%) of these monkeys. The third frequency was Mamu-DQB*1703, which was detected in 29 (24.37%) of these monkeys. Three of the five geographical populations had their unique alleles. Mamu-DQB*1502, Mamu-DQB*1601, Mamu-DQB*170601, Mamu-DQB*1804, and Mamu-DQB*1819 were found only in the Hanyuan population, Mamu-DQB*061301 and Mamu-DQB*1818 were found only in the Danba population, and Mamu-DQB*0607 was found only in the Jiulong population. Mamu-DQB*0614 was found in all populations at different frequency from 1.79% to 21.43%. Mamu-DQB*061301 was detected only in one individual.

### Genetic diversity of the rhesus macaque populations

In order to estimate genetic diversity of the rhesus macaques in western Sichuan based on the adaptive marker, several genetic diversity parameters such as haplotype diversity (*h*), nucleotide diversity (*π*), expected heterozygosity (*He*) and allelic richness (*A*_*R*_) were calculated (Table 2). The values of *h*, *He*, and *A*_*R*_ were 0.718 ~ 0.891, 0.780 ~ 0.875, and 5.733 ~ 11.938, respectively, indicating a relatively high level of genetic diversity at *Mamu-DQB1* locus. The analysis of nucleotide diversity revealed that most of the nucleotide variation was located in the antigen-binding site (ABS) region, and there were at least 20 nucleotide variations on average in each population. The Hanyuan population exhibited the highest genetic diversity: 15 allele haplotypes were observed in 28 animal individuals. The Heishui population exhibited the lowest genetic diversity: only 7 allele haplotypes were observed in 30 animal individuals. Surprisingly, the inbreeding coefficient (*F*_*is*_) in the Hanyuan population was also the highest, whereas it was lowest in Heishui population.

**Table 2:** **Genetic diversity of *Mamu-DQB1 *within rhesus macaque populations**

**Population**	**L**	***h***	***π***	***K***	***Ho***	***He***	***A***_***R***_	***Fis***	**M-*He***	**M-*Rs***
JL	12	0.800	0.07688	20.7564	0.600	0.780	10.275	0.255	0.73	6.27
HY	15	0.891	0.09296	25.1000	0.607	0.875	11.938	0.323	0.72	6.40
DB	8	0.862	0.08600	23.2196	0.786	0.832	8.000	0.092	0.68	5.68
XJ	10	0.807	0.10219	27.5919	0.741	0.792	8.262	0.084	0.70	6.20
HS	7	0.718	0.08387	22.6441	0.833	0.706	5.733	−0.164	0.67	5.27

Notes: L, number of alleles; *h,* haplotype diversity; *π,* nucleotide diversity; *K*: Average number of nucleotide differences; *Ho*, observed heterozygosities; He, expected heterozygosities; *Fis*, the inbreeding coefficient; *A*_*R*_, estimates of allelic richness. M-*He* is the mean heterozygosity of Microsatellite alleles and M-*R*s is the mean allelic number across 30 microsatellite loci per population from Li et al. [25]. JL, Jiulong; HY, Hanyuan; DB, Danba; XJ, Xiaojin; HS, Heishui.

### Phylogenetic analysis

**Figure 3:**
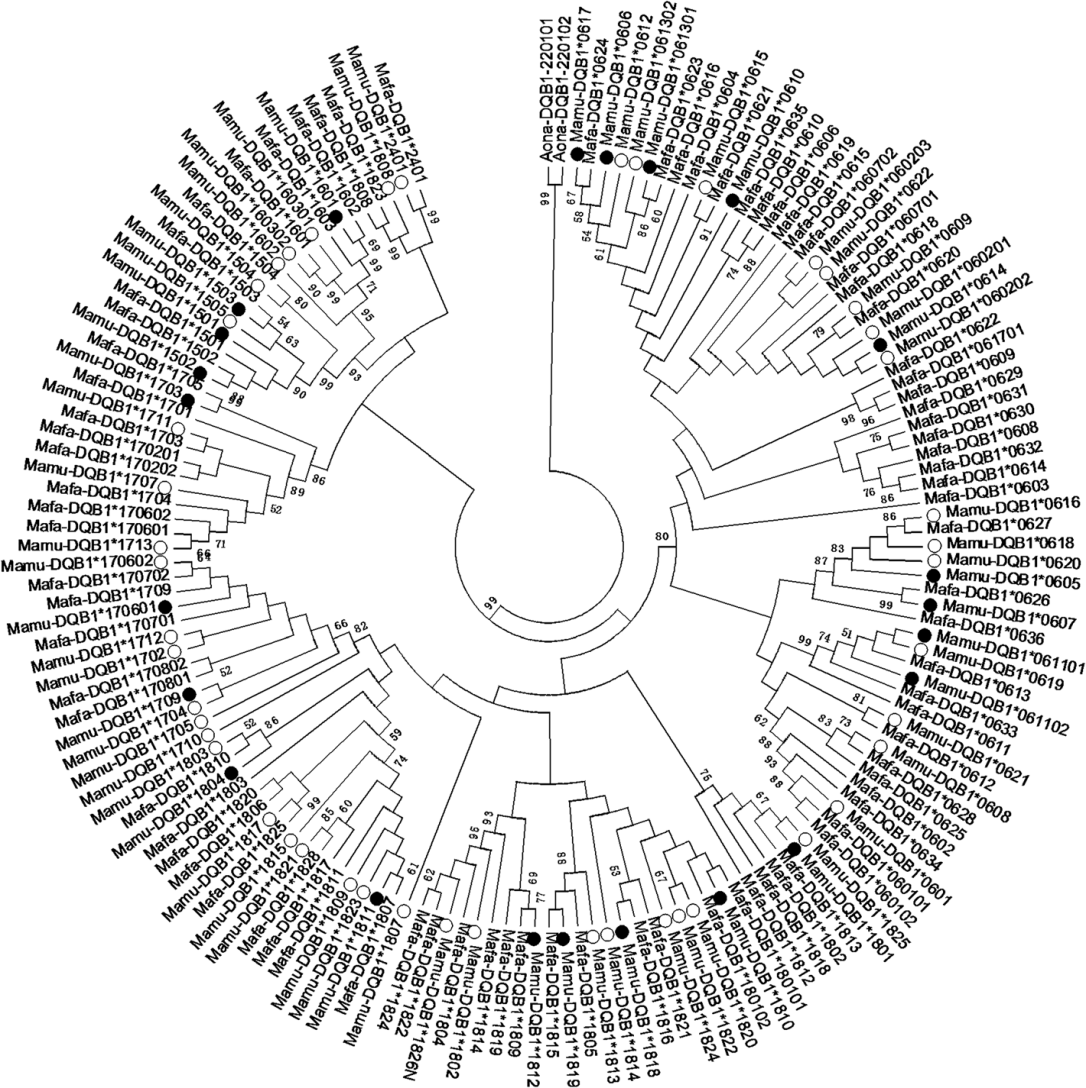
**Phylogenetic tree of the *MhcMamu- and MhcMafa-DQB1 *alleles using neighbour-joing methods.** 23 *Mamu-DQB1* sequences detected in this study are shown with a solid round spot. 46 *Mamu-DQB1* alleles not detected in animals of this study are showed with a circle. 85 *Mafa-DQB1* sequences are showed without anything. 2 *Aona-DQB1* (Aona-DQB1*220101 and Aona-DQB1*220102) were selected as outgroup. Except for 23 *Mamu-DQB1* sequences detected in this study, all other *DQB1* sequences were retrieved from the IPD-MHC Database (http://www.ebi.ac.uk/ipd/mhc/). Numbers at the node are bootstrap value in statistic analysis, only values above 50% are showed.

The phylogenetic tree was constructed for 156 *Mhc-DQB1* gene sequences, including 69 *Mamu-DQB1*, 85 *Mafa-DQB1* and 2 *Aona-DQB1* alleles by using the neighbor-joining method (Figure 3), and *aona-DQB1* alleles were used as the out-group. The 156 sequences can be searched in the IPD-MHC Database (http://www.ebi.ac.uk/ipd/mhc/). As is evident from the tree, the majority of *Mhc-DQB1* alleles tended to cluster together according to lineage group rather than species or sample origins, revealing a trans-species model of evolution. Twenty-three *Mamu-DQB1* alleles were classed into five lineages: DQB1*06, *15, *16, *17, and *18, of which lineage DQB1*06 and *18 were most dominant (9/23 = 39% and 7/23 = 30%, respectively). Almost all previously reported major *Mamu-DQB1* lineages were detected (DQB1*06, *15, *17, *18), except for DQB1*24 lineages that were not found in the western Sichuan macaques examined in this study. It is worth noting that rhesus macaques shared only 14 identical alleles with crab-eating macaques on the phylogenetic tree, but there were 10 alleles to be detected in this study (see Table 1).

### Selective pressure analysis

To analyze the selective pressure on *Mamu-DQB1* exon 2 sequences in western Sichuan rhesus populations, the average non-synonymous and synonymous substitution rates (*d*_*N*_/*d*_*S*_*, ω*) for all sites, antigen-binding sites (ABS), and non-antigen binding sites (non-ABS) were calculated (Table 3, Dataset A: 23 sequences). The highest ω ratio value was found in ABS and was greater than one (*ω* = 1.458), whereas the lowest *ω* ratio value was found in non-ABS and less than one (*ω* = 0.976), but these values did not significantly deviate from the neutral expectation in the *Z*-test (*p* > 0.05). To better understand the substitution pattern of *Mamu-DQB1* exon 2 sequences, 46 *Mamu-DQB1* exon 2 sequences from the previous studies were added to our sequence data to expand our analysis (Table 3, Dataset B: 65 sequences). The same analysis with Dataset A was performed. We found that all *ω* ratio values for dataset B were greater than that of Dataset A and greater than one, especially the *ω* ratio value in the ABS of Dataset B was found to be significantly greater than one (*ω* = 2.041, *p* < 0.05). These results indicated that the ABS of *Mamu-DQB1* exon 2 sequences had been subjected to a strong balancing selection, but the selection signal was not significant in the *Mamu-DQB1* exon 2 sequences of western Sichuan rhesus macaques.

**Table 3:** **Mean numbers of nucleotide substitutions per non-synonymous site (*d***_**N**_**) and synonymous site (*d***_**S**_**) and the d**_**N**_**/d**_**S **_**ratios (ω)**

***Mamu-DQB1***	**Sites**	**n**	***d***_**N**_	***d***_**S**_	**ω**	**P**
Dataset A	ABS	16	0.312 ± 0.073	0.214 ± 0.089	1.458	0.163
	Non-ABS	73	0.082 ± 0.018	0.084 ± 0.021	0.976	0.483
	All	89	0.118 ± 0.020	0.103 ± 0.020	1.146	0.279
Dataset B	ABS	16	0.251 ± 0.081	0.123 ± 0.068	2.041	0.039*
	Non-ABS	73	0.082 ± 0.018	0.078 ± 0.017	1.051	0.427
	All	89	0.106 ± 0.020	0.085 ± 0.017	1.247	0.192

Notes: Dataset A represented 23 *Mamu-DQB1* alleles in this study;Dataset B represented currently 69 *Mamu-DQB1* alleles including 46 alleles from IPD. ABS, the putative amino acid residues involved in antigen binding region (site 9, 11, 13, 28, 47, 57, 61, 67, 70, 71, 74, 78, 85, 86, 89 and 90), were identified according to the corresponding antigen binding sites identified in human HLA-DR and DQ structure (72). n, the number of codons encoding amino acid; Statistical significance is indicated by the asterisks (*P <*0.05).

### Genetic differentiation among five rhesus-macaque populations

From the analysis of molecular variance (AMOVA, Table 4) for the five populations, 85.29% of the total variation in our dataset was observed within populations and 14.71% was observed among populations, showing that the genetic variation primarily came from within population. Although the genetic differentiation within a population was significantly higher than among populations, there was still a moderate differentiation among populations because of a larger fixation index (*F*_ST_) (0.14712, *p* < 0.01). To further assess the genetic differentiation among populations, the pairwise comparison *F*_ST_ and gene flow (*N*m) values among populations were calculated (Table 5). The degree of genetic differentiation was smallest between the Danba and Xiaojin populations, whereas the Heishui and Jiulong populations had the largest differentiation. The Heishui population had relatively little gene exchange with others.

**Table 4:** **Analysis of molecular variance (AMOVA) of *Mamu-DQB1 *sequences in five rhesus macaque populations**

**Source of variation**	**d.f**	**Sum of squares**	**Variance components**	**Percentage of variation**	**Fixation index (*Fst*)**
Among populations	4	14.706	0.06982*Va*	14.71	0.14712*
Within populations	233	94.315	0.40479*Vb*	85.29	
Total	237	109.021			

Notes: *Va*, variance components among populations; *Vb*, variance components within populations; *Significant value (*P* < 0.05).

**Table 5:** Genetic differentiation among five rhesus macaque populations

**Population**	**JL**	**HY**	**DB**	**XJ**	**HS**
JL		3.065	2.473	2.046	1.533
HY	0.075**		4.006	4.255	1.960
	*0.0431*				
DB	0.092**	0.059**		6.175	2.932
	*0.0539*	*0.0599*			
XJ	0.109**	0.055**	0.039*		2.176
	*0.0494*	*0.0573*	*0.0539*		
HS	0.140**	0.113**	0.079**	0.103**	
	*0.0858*	*0.0924*	*0.0976*	*0.0757*	

Notes: Pairwise fixation index (*F*_ST_) values calculated using allele frequency only (lower diagonal), DQB1-*F*_*ST*_ values were in the first row; and microsate- *F*_*ST*_ values were in the second row and were italic (from Li et al., [25]). Gene flow (*N*m) values were in upper diagonal. Statistical significance is indicated by the asterisks (*P < 0.05, **P < 0.01). JL, Jiulong; HY, Hanyuan; DB, Danba; XJ, Xiaojin; HS, Heishui.

**Figure 4:**
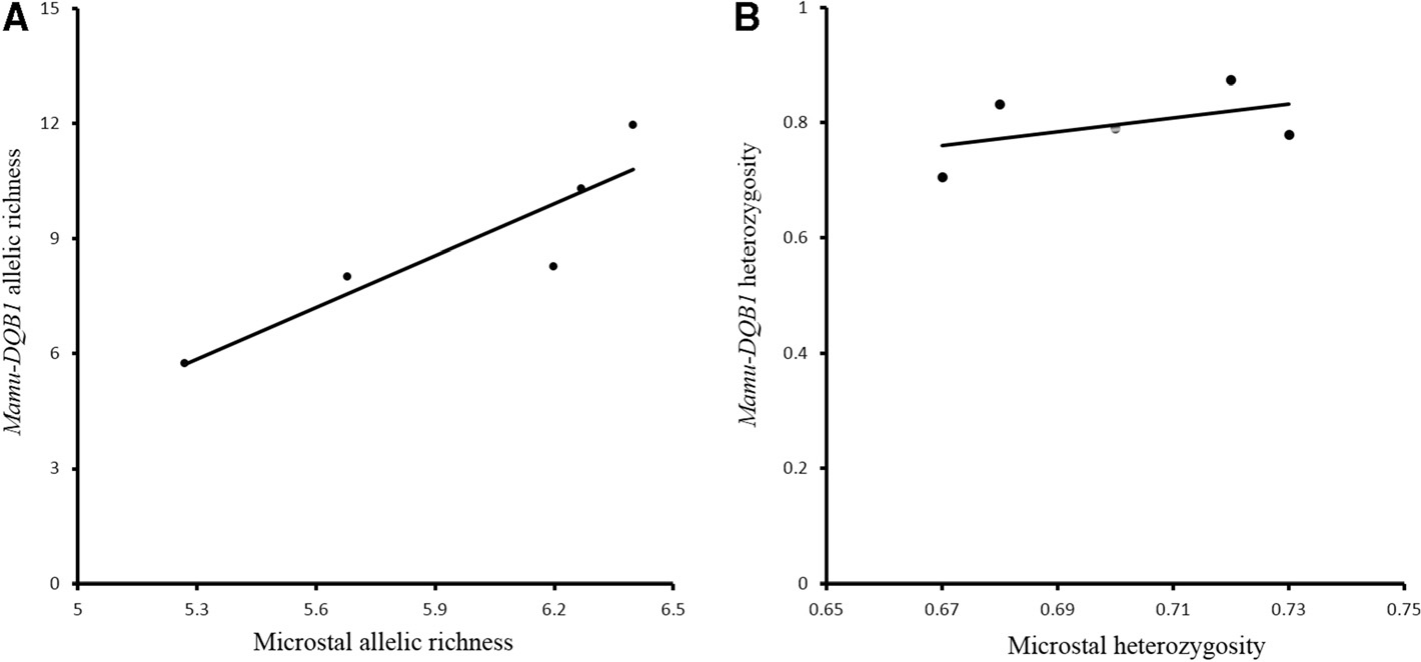
**Correlations between *MhcMamu-DQB1 *allele and microsatellite locus variation. (A)** Mean microsatellite heterozygosity vs. MHC heterozygosity. **(B)** Mean microsatellite allelic richness vs MHC allelic richness per population.

To further explore genetic differentiation, we compared *He* and *A*_*R*_ between microsatellite and *Mamu-DQB1* markers (Figure 4). The richness of MHC sequences per population provides a measure of allelic diversity within each population; the *A*_*R*_ values of both the microsatellite sites and *Mamu-DQB1* are significantly positively correlated (*r*^2^ = 0.961, *df* = 4, *p* = 0.003). However, there is not a significant correlation between microsatellite sites and *Mamu-DQB1* heterozygosity per population (*r*^2^ = 0.2293, *df* = 4, *p* = 0.4145). No significant correlation to pairwise *F*_ST_ between microsatellite sites and *Mamu-DQB1* (*r*^2^ = 0.2198, *df* = 9, *p* = 0.1716). The *F*_ST_ values of *Mamu-DQB1* showed higher divergence among populations when compared with microsatellite loci (except for the Danba-Hanyuan and Danba-Xiaojin populations), and all of the *F*_ST_ values (except for that of the Danba-Xiaojin population) at the *Mamu-DQB1* locus were higher than 0.05, indicating that divergence existed among populations, especially between the Heishui and Jiulong populations, which had the largest *F*_*ST*_. Overally, the population difference was more obvious at the *Mamu-DQB1* locus than the microsatelites: six *F*_*ST*_ values from the *Mamu-DQB1* locus were far larger than that of the microsatellites in ten pariwise population comparisons (Table 5).

## Discussion

### Genetic diversity of *Mamu-MhcDQB1* locus

In various studies, the maintenance of a high level of genetic diversity is assumed to be a preliminary strategy for the population conservation [32-34]. More specifically, high genetic diversity has often been shown to be positively correlated with indicator values of individual fitness such as the mating success of animals and adaptability to changing environmental conditions [35,36]. In accordance with a previous study in rhesus monkeys, Chinese-origin rhesus macaques have a greater genetic variation at the *Mamu-DQB1* locus and higher nucleotide diversity of mtDNA when compared with Indian-origin rhesus macaques [31,37]. For example, only 16 *Mamu-DQB1* alleles were identified from 107 Indian origin individuals born in captivity [38], whereas 23 *Mamu-DQB1* alleles were identified from 119 Chinese rhesus macaques in this study.

The high level of polymorphism usually observed in MHC genes is likely to be maintained by balancing selection, driven largely by exposure to a diversity of pathogens [9-11]. In the present study, the Hanyuan population had conspicuously higher *A*_*R*_, haplotype diversity (*h*) and *He* values than any other populations (Table 2), exhibiting higher genetic diversity. This implied a strong balancing selection force that may have occurred in this population. In addition, the Hanyuan population lives in the Xiangling Mountains which is a transition zone between the north and south of two distinct climates, the wet and cold Qionglai mountains in the north and the dry and hot Liangshan mountains in the south. Thus, the climate transition zone might also be a genetic intersection area of species, leading to higher genetic diversity than other nearby areas. However, the Heishui population exhibited a lower level of genetic diversity than other populations. In particular, Mamu-DQB1*0614 and Mamu-DQB1*1703 are significantly more frequent in this population (Table 1). It is implied that a founder effect or genetic drift may have occurred in Heishui population. In addition, previous analyses of *MHC* variation in species that span large geographical ranges have emphasized the role of diversifying selection on MHC genes [39-42], thus we can not rule out the possibility of strong selection for the two alleles (Mamu-DQB1*0614 and Mamu-DQB1*1703) that are present at considerably higher frequencies in this population. Nevertheless, it is possible that there are additional *MHC* alleles that were not detected in Heishui population because of its smaller sample size.

Disassortative mate choice may also contribute to the maintenance of MHC diversity [43,44]. Choosing partners with maximal or optimal genetic dissimilarity may help to avoid inbreeding or excessive outbreeding, respectively. To increase offspring diversity at key functional loci in a natural population, a recent study indicated that inbreeding avoidance was along with disassortative mate choice at *DRB*, but not at *DQB* locus [45]. Our results also seem to support this point. For example, the Hanyuan and Jiulong populations had high levels of genetic diversity, but they also had the highest inbreeding coefficients (*Fis*) at the *Mamu-DQB1* locus (Table 2).

### Balancing selection and trans-species polymorphism

Balancing selection is expected to preserve high levels of polymorphisms at MHC loci by retaining alleles during species diversification events [46,47]. The *d*_*N*_ /*d*_*S*_ test for high rates of non-synonymous (*d*_*N*_*)* compared to synonymous (*d*_*S*_*)* substitutions (*ω* = *d*_*N*_ /*d*_*S*_*)* is the most common measure used to detect balancing selection acting on protein-coding genes. Polymorphisms within the ABS of MHC genes are thought to be maintained by balancing selection imposed by pathogens/parasites. Fast-evolving parasites may adapt to the most common host genotype and escape presentation of their antigens to the adaptive immune system of the host [48]. In this study, the analysis of the nucleotide acid substitution pattern showed that *Mamu-DQB1* exon 2 sequences had been subjected to a strong balancing selection, but the selection signal was not significant in the *Mamu-DQB1* exon 2 sequences of western Sichuan rhesus macaques. This may well be caused by genetic drift acting differently on the different populations, e.g., the Heishui population may has been subject to a genetic drift, leading to a low level of genetic diversity. Alternatively, this may also be due to the smaller sample size surveyed here.

Under balancing selection, some allelic lineages exhibit unusual longevity that predates speciation events, leading to “trans-species polymorphisms” (TSP) used to describe polymorphisms among species [49]. In an extreme case of TSP, different species share identical alleles. Identical MHC gene sequences can be found in different species that have been separated by millions of years of independent evolution [50]. This is remarkable, given that these genes are involved in a co-evolutionary arms race with parasites [51]. In the present study, the phylogenetic analysis has illustrated that *Mhc-DQB1* allele sequences from rhesus and cynomolgus macaques tended to cluster together according to allelic lineages rather than species or sample origins, leading to the sharing of allelic lineages by different species. The result clearly confirmed the trans-species model of evolution of the *Mhc-DQB1* lineages. In particular, 14 *Mamu-DQB1* alleles matched exactly with the *Mafa-DQB1* alleles, suggesting that they may have occurred because of trans-species conservation after the divergence of the rhesus and cynomolgus macaque 1.8–2.0 million years ago (mya) [52,53,28]. Alternatively, the identical nucleotide sequences in the rhesus and cynomolgus macaques may have arisen by occasional interbreeding between the two species. In fact, a recent study on genome sequencing and comparison of the cynomolgus and Chinese rhesus macaques showed that the cynomolgus macaque genome has been shaped by introgression after hybridization with the Chinese rhesus macaque [54]. The allele sharing between the rhesus and cynomolgus macaques has also been found in other MHC class II genes such as *DQA1*, *DPB1*, and *DRB1*[38]. However, it is unusual that the sharing phenomenon has not been by now detected in any MHC class I genes, which is a synthetic group in the same chromosome with class II genes.

### Genetic differentiation among populations

AMOVA of the five populations indicated that variations mainly occurred among populations and that the *F*st was moderate (0.14712) (Table 4). *F*st represents the level of genetic differentiation among populations [55,56]: an *F*st of 0 to 0.05 represents “little differentiation,” 0.05 to 0.25 “moderate differentiation,” and values greater than 0.25 “very great differentiation” [57]. Therefore, our results revealed that moderate genetic differentiation had occurred among the five rhesus macaque populations. To further assess genetic differentiation among the five populations, we also estimated *F*st and *Nm. Nm* (also known as gene migration) is the transfer of genes from one population to another. Generally, if *N*m < 1, genetic drift will result in substantial local differentiation; if *N*m > 1, gene flow between populations is higher and the extent of genetic differentiation is smaller. When *Nm* > 4, gene exchange is more frequent and genetic differentiation is much smaller [58,59]. In this study, all values of *Nm* were larger than 1 (1.533–6.175). Especially, the values of *Nm* among three populations (Danba, Xiaojin, and Hanyuan) were even larger than 4, suggesting that gene exchange was more frequent among the three populations.

One hallmark of animal mtDNA is strict maternal inheritance. Previous study about mtDNA indicated poor gene exchange occurred in these five populations with the *N*m values less than 1, thus gene exchange may occur mainly in nuclear genes. In other words, genetic exchanges mainly come from the migration of paternal macaques, which is consistent with male-biased migration behavior in various macaques. However, the levels of differentiation among the five populations were moderate. The differentiation were highest between the Jiulong and Heishui populations, and lowest between the Danba and Xiaojin populations. We speculated that genetic differentiation between these populations has arisen due to the geographical barrier of the high mountains, large rivers and long geographic distances between populations. For example, Jiulong and Danba populations are located between the Yalongjiang and Dadu Rivers, but they are separated by the Gongga Mountains. It is difficult for rhesus macaques to cross these geographical barriers because of their vast width, depth, length, and flow. In fact, it has been shown that large rivers formed a natural barrier that obstructs gene exchange between many mammal populations [60,61].

Differences in parasite abundance and diversity in environments may result in selection for different sets of alleles in different populations. Loci under balancing selection are predicted to own a higher variation within population, but lower differentiation between populations when compared with neutral or nearly neutral markers, assuming that the selective pressures in the two populations are similar [62]. However, genes under directional selection present increased divergence between populations relative to neutral or nearly neutral markers if selective pressures differ between populations, or they may present decreased divergence relative to neutral or nearly neutral markers if selection pressures are similar [63]. Thus, compared with neutral forces, balancing selection is supposed to diminish population differentiation as measured by conventional pairwise *F*_*ST*_. Hence, the population structure under balancing selection should not be distinct. In the present study, nine out of ten of the pairwise *F*_*ST*_ values of *Mamu-DQB1* were greater than 0.05 and eight pairwise *F*_ST_ values were even greater than that of the microsatellites (Table 2). A significant positive correlation was found between allelic richness in *Mamu-DQB1* and microsatellites; a similar positive correlation was also found on pairwise *F*_*ST*_ values, implying that genetic drift had played a significant role in maintaining MHC diversity for rhesus macaques. However, as mentioned above, the sharing of allelic lineages between species observed on our phylogenetic tree indicated a “trans-species polymorphism” under balancing selection, but the rhesus and cynomolgus macaques have diverged from each only 1.8–2.0 mya [53]. Therefore, the balancing selection acting on *Mamu-DQB1* may be historical.

## Conclusion

It is widely accepted that variation at MHC loci is maintained by balancing selection, even with a low level of neutral variability in some species [64,65], indicating the importance of balancing selection for maintaining genetic variation in the wild and also exposing the limitations of the neutral marker as substitutes for variation in fitness-related genes [66]. However, balancing selection may be overwhelmed by other microevolutionary forces such as genetic drift in small and isolated or bottlenecked populations [67]. In this study, we detected evidence of a weaker balancing selection and found similar genetic variation pattern both at neutral and adaptive markers, suggesting that genetic drift was stronger than selection, thus leading to a reduction of MHC diversity in the local populations. AMOVA results also revealed that a moderate genetic differentiation had occurred in several segregated populations in western Sichuan, China, due to habitat fragmentation caused by long-term geographic barriers and human activities. The Heishui population should be paid more attention for its lowest level of genetic diversity and relatively high divergence from others. This study provides deep insight into genetic variation of rhesus macaque populations in the fragmented habitat and helps to make an effective strategy for species conservation and facilitate the use of rhesus macaque in biomedical research. However, we have to acknowledge that the findings in this study are still very limited because of the smaller sample size as well as a single *MHC* gene locus. Therefore, to clarify thoroughly the genetic variation pattern of rhesus macaque populations in western China, further research is still needed using other *MHC* genes, other immune system loci and other markers, as well as more comprehensive sampling.

## Methods

### Samples and Genomic DNA extraction

The whole blood samples were collected from 119 western Sichuan rhesus macaques at the National Experimental Rhesus Research Centre of Sichuan Agricultural University. All of these animals were the first-captured founder population from five different geographical populations in the wild, Jiulong (n = 20), Hanyuan (n = 28), Danba (n = 14), Xiaojin (n = 27), and Heishui (n = 30), located in western Sichuan, China (Figure 1). For each population, the samples are selected randomly to try to ensure that no correlation existed between animal individuals, i.e., they were not from the same family. All samples were collected in strict compliance with the Chinese Wildlife Conservation Act. Also, the Sichuan Agricultural University Ethics Committee provided ethical approval for this study. Genomic DNA was extracted from samples according to the standard phenol-chloroform method [68]. Products were examined with 1.0% agarose gel electrophoresis and were visualized under ultraviolet light.

### PCR and SSCP typing

A pair of specific primers (Mamu-DQB1F 5’–GCCTGACTGACTGGACGGTGATTC-3’ and Mamu-DQB1R 5’-GGGGCGACAACGCTCACCTC-3’) were used to amplify an approximately 317-bp DNA fragment containing the complete second exon of the *Mamu-DQB1* gene. For polymerase chain reaction (PCR) amplification of *Mamu-DQB1*, all PCRs were performed in a reaction volume of 10 μl, containing 5 μl of 2X ES Taq MasterMix (CWBIO, Beijing, China), 0.3 μl (10 pm/μl) of each *DQB1* primer, roughly 20 ng template DNA, and distilled, deionized H_2_O to 10 μl. All PCR amplifications were carried out using a MyCycler^TM^ Thermal Cycler (BIO-RAD, USA), with the first step at 94°C for 5 minutes, 30 cycles of 94°C for 30 seconds, 62°C for 25 seconds, 72°C for 45 seconds, and a final extension at 72°C for 10 minutes. Products were examined with 1% agarose gel electrophoresis and visualized under ultraviolet light.

All positive PCR amplification products were screened for polymorphisms in *DQB1* exon 2 by SSCP analysis. Two μl of the PCR sample was mixed with 8 μl of the denaturating buffer (95% formamide, 5% 6X loading buffer) before heating for 10 min at 98°C. The samples after denaturation were immediately cooled in ice water at least 10 min, then loaded in a 2-mm-thick 12% acrylamide gel (no glycerol contained) and were electrophorized in 0.5X TBE at 130 V (15 h) at room temperature. The resulting bands were visualized by silver staining. PCR products that showed the same SSCP pattern were counted and typed.

### Cloning and sequencing

Representative samples from each population were amplified with a reaction volume of 50 μl by PCR; the product was fractionated by electrophoresis in 18% agarose gels and purified using a Gel Extraction Kit (Sangon Biotech, Shanghai, China). Purified pieces of DNA were then cloned using the pMD19-T Vector kit (Takara, Dalian, China). Briefly, ~2–3 μl of each positive purified DNA was added into each mixture containing 2.5 μl of Solution I and 0.5 μl of pMD19-T Vector. The mixtures were incubated at 16°C for 40 min. For transformation, we added 25 μl of *Escherichia coli* DH5α-competent cells to the incubated ligation mixture, and then we put this on ice for 30 minutes, heat-shocked the cells at 42°C for 52 seconds in a water bath, immediately put this on ice for at least 3 minutes, and added 800 μl 37°C LB media. This mixture was then incubated at 37°C with shaking at approximately 150 rpm for 80 min, and recovery cells were mixed with 7 μl IPTG and 40 μl X-Gal, then 160 μl of the mixture was plated onto LB/ampicillin plates and incubated at 37°C overnight (14–16 hours). After blue⁄white selection, a number of positive clones were picked and grown, and 10–12 clones were chosen and sequenced by the sequencing company (Invitrogen, Shanghai, China). To avoid errors arising from PCR-based recombination, apart from the optimization of the PCR program, we have taken several measures to determine a real sequence: 1) a sequence must be detected in at least 2 animal individuals; and/or 2) if a sequence was found in only one animal individual, it must be confirmed by at least 2 independent PCRs.

### Data analysis

#### *Mamu-DQB1* diversity analyses

The sequences obtained from this study were initially adjusted using DNAStar software (DNASTAR Inc., Madison, WI). Multiple sequence alignments were created using the Clustal W program in DAMBE software [69]. Nucleotide diversity (*π*) and haplotype diversity (*h*) were calculated using DnaSP (Version 5) [70]. Allelic frequency distribution, expected (*He*) and observed *(Ho*) heterozygosities, pairwise *F*_ST_ and genetic flow (*N*m) among populations were estimated with GenAlEx 6.5 [71]. Inbreeding coefficient (*F*_*IS*_) and allelic richness (*A*_*R*_) per population were calculated with the FSTAT program, version 2.9.3 [72]. Allelic richness is a measure of the number of alleles independent of sample size. Hence, it can be compared between samples of different sizes.

### Selection pressure and phylogenetic analysis

Non-synonymous (*d*_*N*_*)* and synonymous (*d*_*S*_*)* substitution rates at *Mamu-DQB1* amino acid sites were calculated according to the Nei–Gjobori method in Mega 5.05 [73]; the *Z-*test for selection was used to evaluate whether *d*_*N*_ was significantly greater than *d*_*S*_ for entire sequences. The ABS and non-ABS were distinguished based on *HLA-DQB1* structure [74]. Phylogenetic trees were reconstructed for *Mhc-DQB1* allelic sequences using Mega 5.05, based on the neighbor-joining method with Kimura 2-para model. The reliability of the neighbor-joining tree was estimated by bootstrapping, in which 1000 bootstrap pseudosamples were used. To expand our data and compare with the closely related species, an additional 46 *Mamu-DQB1*, 85 *Mafa-DQB1* (*Macaca fascicularis*), and 2 *Aona-DQB1* (*Aotus nancymaae*) alleles were also retrieved from the Immuno Polymorphism Database. Thus, a total of 156 *Mhc-DQB1* gene sequences including 23 sequences identified in this study were used in the phylogenetic analysis, and *Aona-DQB1* was used as an out-group. Information for all these sequences is shown in Additional file 1: Table S1.

### *Mamu-DQB1* genetic differentiation analyses

The degree of population subdivision was estimated on the basis of hierarchical analysis of molecular variance (AMOVA) using the Arlequin version 3.1 package [75]. This package was used to compare the component of genetic diversity from the variance among the sampled major geographical regions and among subpopulations within each region. In order to measure the role of balancing selection in maintaining MHC variation among the population, the microsatellite data sets of Li et al. [25] that analyzed microsatellite variation at 30 loci in six populations (including all populations in this study) were used, and correlations between *Mhc-DQB1* and microsatellite data were assessed using the Pearson product–moment correlation in SPSS 16.0.

## Availability of supporting data

The data sets supporting the results of this article are available in the Dryad repository, doi:10.5061/dryad.h8b93 [76].

## Competing interests

The authors have declared that no competing interests.

## Authors’ contributions

H-LX conceived the study and helped to draft the manuscript; Y-FY and Q-XD performed the experiments and wrote the manuscript; JL, Q-YN and M-WZ analyzed the data. All authors read and approved the final manuscript.

## Supplementary Material

Additional file 1: Table S1 *Mhc-DQB1* alleles of different primate species used in this study A total of 156 *Mhc-DQB1* alleles from three primate species (*Macaca mulatta*, *Macaca fascicularis*, *Aotus nancymaae*) were used in this study, including 69 *Mamu-DQB1*, 85 *Mafa-DQB1* and 2 *Aona-DQB1* alleles. Alleles detected in this study are shown in bold. IPD Acc No. is accession number in the IPD-MHC Database (http://www.ebi.ac.uk/ipd/mhc/). Click here for file
