# Supplementary material for: Genetic diversity and differentiation of the rhesus macaque (Macaca mulatta) population in western Sichuan, China, based on the second exon of the major histocompatibility complex class II DQB (MhcMamu-DQB1) alleles
Source: BMC Evol Biol. 2014 Jun 14;14:130. doi: 10.1186/1471-2148-14-130 (PMC4070090; doi:10.1186/1471-2148-14-130)
Supplement: Additional file 1: Table S1 — Mhc-DQB1 alleles of different primate species used in this study A total of 156 Mhc-DQB1 alleles from three primate species (Macaca mulatta, Macaca fascicularis, Aotus nancymaae) were used in this study, including 69 Mamu-DQB1, 85 Mafa-DQB1 and 2 Aona-DQB1 alleles. Alleles detected in this study are shown in bold. IPD Acc No. is accession number in the IPD-MHC Database (http://www.ebi.ac.uk/ipd/mhc/). [file 1471-2148-14-130-S1.pdf]

Table S1. *Mhc-DQB1* alleles of different primate species used in this study

Allele name	IPD Acc No[1]	Allele name	IPD Acc No[1]
*Mamu-DQB1*06:01*	MHC00398	*Mafa-DQB1*06:09*	MHC00285
*Mamu-DQB1*06:02:01*	MHC00399	*Mafa-DQB1*06:10*	MHC00286
*Mamu-DQB1*06:02:02*	MHC03494	*Mafa-DQB1*06:11*	MHC00287
*Mamu-DQB1*06:02:03*	MHC04024	*Mafa-DQB1*06:12*	MHC00288
Mamu-DQB1*06:05	MHC00400	*Mafa-DQB1*06:13*	MHC00289
Mamu-DQB1*06:06	MHC00401	*Mafa-DQB1*06:14*	MHC00290
Mamu-DQB1*06:07	MHC00402	*Mafa-DQB1*06:15*	MHC01188
*Mamu-DQB1*06:08*	MHC00403	*Mafa-DQB1*06:16*	MHC03518
*Mamu-DQB1*06:09*	MHC00404	*Mafa-DQB1*06:17:01*	MHC03519
Mamu-DQB1*06:10	MHC00405	*Mafa-DQB1*06:18*	MHC03521
Mamu-DQB1*06:11:01	MHC00406	*Mafa-DQB1*06:19*	MHC03522
Mamu-DQB1*06:11:02	MHC00407	*Mafa-DQB1*06:20*	MHC03523
*Mamu-DQB1*06:12*	MHC00408	*Mafa-DQB1*06:21*	MHC03524
Mamu-DQB1*06:13:01	MHC00409	*Mafa-DQB1*06:22*	MHC03525
*Mamu-DQB1*06:13:02*	MHC02196	*Mafa-DQB1*06:23*	MHC03526
Mamu-DQB1*06:14	MHC01626	*Mafa-DQB1*06:24*	MHC03527
*Mamu-DQB1*06:15*	MHC02192	*Mafa-DQB1*06:25*	MHC03528
*Mamu-DQB1*06:16*	MHC02350	*Mafa-DQB1*06:26*	MHC03529
Mamu-DQB1*06:17	MHC02905	*Mafa-DQB1*06:27*	MHC03698
*Mamu-DQB1*06:18*	MHC03495	*Mafa-DQB1*06:28*	MHC03699
*Mamu-DQB1*06:19*	MHC03496	*Mafa-DQB1*06:29*	MHC03700
*Mamu-DQB1*06:20*	MHC04013	*Mafa-DQB1*06:30*	MHC03701
*Mamu-DQB1*06:21*	MHC04014	*Mafa-DQB1*06:31*	MHC03777
*Mamu-DQB1*06:22*	MHC04015	*Mafa-DQB1*06:32*	MHC03779
Mamu-DQB1*15:01	MHC00410	*Mafa-DQB1*06:33*	MHC03888
Mamu-DQB1*15:02	MHC00411	*Mafa-DQB1*06:34*	MHC03889
Mamu-DQB1*15:03	MHC00412	*Mafa-DQB1*06:35*	MHC04715
*Mamu-DQB1*15:04*	MHC02193	*Mafa-DQB1*06:36*	MHC05121
*Mamu-DQB1*15:05*	MHC04025	*Mafa-DQB1*15:01*	MHC00291
Mamu-DQB1*16:01	MHC00413	*Mafa-DQB1*15:02*	MHC00292
*Mamu-DQB1*16:02*	MHC00414	*Mafa-DQB1*15:03*	MHC00293
*Mamu-DQB1*16:03:01*	MHC00415	*Mafa-DQB1*15:04*	MHC03778
*Mamu-DQB1*16:03:02*	MHC03880	*Mafa-DQB1*16:01*	MHC00294
*Mamu-DQB1*17:02*	MHC00416	*Mafa-DQB1*16:02*	MHC03447
Mamu-DQB1*17:03	MHC00417	*Mafa-DQB1*16:03*	MHC03697
*Mamu-DQB1*17:04*	MHC00418	*Mafa-DQB1*17:01*	MHC00295
*Mamu-DQB1*17:05*	MHC00419	*Mafa-DQB1*17:02:01*	MHC00296
Mamu-DQB1*17:06:01	MHC00420	*Mafa-DQB1*17:02:02*	MHC04132
*Mamu-DQB1*17:06:02*	MHC03500	*Mafa-DQB1*17:03*	MHC00297
*Mamu-DQB1*17:07*	MHC00421	*Mafa-DQB1*17:04*	MHC00298
Mamu-DQB1*17:09	MHC00423	*Mafa-DQB1*17:05*	MHC00299
*Mamu-DQB1*17:10*	MHC01277	*Mafa-DQB1*17:06:01*	MHC03445
*Mamu-DQB1*17:11*	MHC02194	*Mafa-DQB1*17:06:02*	MHC03446
*Mamu-DQB1*17:12*	MHC03499	*Mafa-DQB1*17:07:01*	MHC03438
*Mamu-DQB1*17:13*	MHC04026	*Mafa-DQB1*17:07:02*	MHC03513
Mamu-DQB1*18:01	MHC00424	*Mafa-DQB1*17:08:01*	MHC03516
*Mamu-DQB1*18:02*	MHC00425	*Mafa-DQB1*17:08:02*	MHC03661
*Mamu-DQB1*18:03*	MHC00426	*Mafa-DQB1*17:09*	MHC03866
Mamu-DQB1*18:04	MHC00427	*Mafa-DQB1*18:01:01*	MHC00300
*Mamu-DQB1*18:07*	MHC00428	*Mafa-DQB1*18:01:02*	MHC03444
*Mamu-DQB1*18:08*	MHC00429	*Mafa-DQB1*18:02*	MHC00301
*Mamu-DQB1*18:09*	MHC00430	*Mafa-DQB1*18:03*	MHC00302
Mamu-DQB1*18:10	MHC00431	*Mafa-DQB1*18:04*	MHC00303
Mamu-DQB1*18:11	MHC00432	*Mafa-DQB1*18:05*	MHC00304

Mamu-DQB1*18:12	MHC00433	*Mafa-DQB1*18:06*	MHC00305
*Mamu-DQB1*18:13*	MHC00434	*Mafa-DQB1*18:07*	MHC00306
*Mamu-DQB1*18:14*	MHC00435	*Mafa-DQB1*18:08*	MHC00307
*Mamu-DQB1*18:15*	MHC01067	*Mafa-DQB1*18:09*	MHC03439
*Mamu-DQB1*18:17*	MHC02191	*Mafa-DQB1*18:10*	MHC03440
Mamu-DQB1*18:18	MHC02195	*Mafa-DQB1*18:11*	MHC03441
Mamu-DQB1*18:19	MHC02197	*Mafa-DQB1*18:12*	MHC03442
*Mamu-DQB1*18:20*	MHC02198	*Mafa-DQB1*18:13*	MHC03443
*Mamu-DQB1*18:21*	MHC02199	*Mafa-DQB1*18:14*	MHC03512
*Mamu-DQB1*18:22*	MHC02906	*Mafa-DQB1*18:15*	MHC03514
*Mamu-DQB1*18:23*	MHC03497	*Mafa-DQB1*18:16*	MHC03515
*Mamu-DQB1*18:24*	MHC03498	*Mafa-DQB1*18:17*	MHC03517
*Mamu-DQB1*18:25*	MHC03578	*Mafa-DQB1*18:18*	MHC03702
*Mamu-DQB1*18:26N*	MHC04027	*Mafa-DQB1*18:19*	MHC03703
*Mamu-DQB1*24:01*	MHC00436	*Mafa-DQB1*18:20*	MHC03776
*Mafa-DQB1*06:01:01*	MHC00278	*Mafa-DQB1*18:21*	MHC03780
*Mafa-DQB1*06:01:02*	MHC01623	*Mafa-DQB1*18:22*	MHC03863
*Mafa-DQB1*06:02*	MHC00279	*Mafa-DQB1*18:23*	MHC03864
*Mafa-DQB1*06:03*	MHC00280	*Mafa-DQB1*18:24*	MHC03865
*Mafa-DQB1*06:04*	MHC00281	*Mafa-DQB1*18:25*	MHC04240
*Mafa-DQB1*06:06*	MHC00282	*Mafa-DQB1*18:28*	MHC05119
*Mafa-DQB1*06:07:01*	MHC00283	*Mafa-DQB1*24:01*	MHC00308
*Mafa-DQB1*06:07:02*	MHC03520	*Aona-DQB1*22:01:01*	MHC00006
*Mafa-DQB1*06:08*	MHC00284	*Aona-DQB1*22:01:02*	MHC00007

Notes: A total of 156 *Mhc-DQB1* alleles from three primate species (*Macaca mulatta*, *Macaca fascicularis*, *Aotus nancymaae*) were used in this study, including 69 *Mamu-DQB1*, 85 *Mafa-DQB1* and 2 *Aona-DQB1* alleles. Alleles detected in this study are shown in bold. [1]IPD Acc No is accession number in the IPD-MHC Database (http://www.ebi.ac.uk/ipd/mhc/).
